# Comparison of two lipid emulsions on interleukin-1β, interleukin-8 and fatty acid composition in infants post gastrointestinal surgery: a randomized trial

**DOI:** 10.12688/f1000research.26269.3

**Published:** 2020-12-21

**Authors:** Meta Herdiana Hanindita, Roedi Irawan, I Dewa Gede Ugrasena, I. G. B. Adria Hariastawa

**Affiliations:** 1 Doctoral Program, Faculty of Medicine, Universitas Airlangga, Surabaya, East Java, 60286, Indonesia; 2 Department of Child Health, Faculty of Medicine, Universitas Airlangga, Surabaya, East Java, 60286, Indonesia; 3 Department of Pediatric Surgery, Faculty of Medicine, Universitas Airlangga, Surabaya, East Java, 60286, Indonesia

**Keywords:** Parenteral Nutrition, Intravenous Lipid Emulsion, Interleukin-1Beta, Interleukin-8, Omega-3

## Abstract

**Background:** Nutritional support plays an essential role for recovery in infants who undergo gastrointestinal surgery. The current standard type of intravenous lipid emulsion (IVLE) used as parenteral nutrition is the mixture of medium-chain triglyceride (MCT) and long chain triglyceride (LCT) rich in ω-6. Studies showed that ω-6 is associated with higher level of proinflammatory cytokines, leading to increased mortality rate, morbidity rate, and postoperative recovery time. The latest generation of emulsion is a mixture of MCT, LCT, olive oil (OO), and fish oil (FO) which may optimize the ω6/ω3 ratio. This study aimed to compare the effect of MCT/LCT/OO/FO IVLE to standard IVLE on IL-1β, IL-8 and serum fatty acids in infants who had undergone gastrointestinal surgery.

**Methods:** A single-blind, randomised controlled, pretest-posttest design study was done in twelve subjects that were classified into two groups. Group 1 received standard IVLE, group 2 received MCT/LCT/OO/FO IVLE. The type of standard and MCT/LCT/OO/FO IVLE used in this study were Lipofundin 20% and SMOFlipid 20%, respectively, both administered for three consecutive days in 1-4 gram/kilogram/day. IL-1β and IL-8 were examined using ELISA while fatty acids was analyzed using gas chromatography tandem mass spectrometry (GC-MS). Statistical analyses were performed using SPSS for Mac 23.

**Results:** No statistical difference was found in age, gender, birth weight and diagnosis between both groups. Leukocyte was significantly lower in MCT/LCT/OO/FO group 3 days after surgery (p=0.025). CRP was lower in MCT/LCT/OO/FO group 3 days after surgery (p=0.01) and in changes within 3 days (p=0.016). There were no differences in IL-1β, IL-8 and ω-3 but ω-6 was higher in standard IVFE group on third day after surgery (p=0,048)

**Conclusion:** MCT/LCT/OO/FO IVLE can significantly lower leukocyte, CRP and ω-6 levels and is comparable with standard IVLE on IL-1β, IL-8 and ω-3 levels in infants who had undergone gastrointestinal surgery.

## Introduction

Surgical interventions may stimulate physiological inflammatory response as body’s attempt towards general recovery ^1^. The balance of inflammatory response brings about good recovery, while excessive level of proinflammatory cytokines such as interleukin (IL) )-1β, IL-6, IL-8, and tumor necrosis factor (TNF)-α, may cause organ damage and severe complications, leading to the rise of postoperative mortality and morbidity rate ^2^. De Mooij stated that IL-1β plays an important role in infection control, homeostasis, and tissue repair, while IL-8 plays an important role in inflammation and wound healing ^3– 5^.

Nutritional support is essential in wound healing and plays an important role in growth and development of an infant after undergoing gastrointestinal surgery ^6^. Patients who could not receive oral and enteral nutrition for two days should be considered for parenteral nutrition ^7, 8^. The current standard type of fat emulsion used as parenteral nutrition is a mixture of medium-chain triglyceride (MCT) and soy oil enriched with long-chain triglyceride (LCT) ^9^. This emulsion is rich in ω-6 fatty acids and contains linoleic acid (LA, C18:2 ω -6) and also α-linolenic acid (ALA, C18:3 ω-3). Several studies showed that ω-6 fatty acids are associated with impaired cell-mediated immunity and higher potential risk of elevated proinflammatory biomarkers and severe inflammatory response. These mechanisms may bring about the rise in mortality rate, morbidity rate and may also prolong the duration of treatment and postoperative recovery time ^10– 12^.

The latest generation of fat emulsion, SMOFlipid, is a mixture of MCT, LCT, olive oil (OO), and fish oil (FO), optimizing the ω6/ ω3 ratio. Some studies showed that OO exerts indirect anti-inflammatory effect by replacing ω-6 fatty acids with oleic acid, while the addition of ω-3 fatty acids from FO in soy-oil based fat emulsion may inhibit inflammatory reactions, i.e. reducing cytokine secretion and adhesion molecule expression and balancing the immune system, since it contains eicosapentaenoic acid (EPA) and docosahexaenoic acid (DHA) ^6, 13– 16^. ω-3 fatty acids may also act as a regulator of the immune system and mitigator of inflammation since it acts as a precursor for lipid mediator ^16^. Our previous study showed lower levels of proinflammatory cytokines IL-6 and TNF-α in subjects who received the mixture of MCT/LCT/OO/FO compared with subjects who received MCT/LCT IVLE ^17^. To our knowledge, there been no study regarding the effects of MCT/LCT/OO/FO IVLE on IL-1β and IL-8 levels and fatty acid composition in infants who undergo gastrointestinal surgery, compared to those who receive standard IVLE.

## Methods

### Study background and recruitment

This single-blind, randomized controlled, pretest-posttest design, parallel-group with 1:1 randomization study aimed to compare the effect of MCT/LCT/OO/FO IVLE to standard MCT/LCT IVLE on IL-1β, IL-8 levels and fatty acid composition in infants who had undergone gastrointestinal surgery. The primary outcomes of this study were IL-1β and IL-8 levels and fatty acid composition while the secondary outcomes were hemoglobin, leukocyte, C-reactive protein (CRP) and albumin levels. This study was conducted in April–July 2020. Our subjects were infants who had undergone gastrointestinal surgery at Soetomo General Hospital, Surabaya. Parents or legal guardian were recruited to the study through referrals from physicians who then contacted the research team. The number of subjects was determined based on the formula for calculating number of samples in non-comparative numerical analytical study, with type 1 error of 5% and type 2 error of 10%, and the minimum number samples for each group were 5 subjects ^18^. The effect size that was used in this formula was 20 pg/ml, with standard deviation of 10.6 pg/ml. This effect size and standard deviation were used to measure our primary outcome (Interleukin-1β). Inclusion criteria for this study included subjects whose parents were willing for them to participate in this study, had undergone gastrointestinal surgery, and had received parenteral nutrition for at least 3 days. Exclusion criteria included subjects who had chronic diseases and subjects who were allergic to fish, egg, soy and/or nut proteins. Adverse effects were minimal or rare. However, if any harm was seen in the subjects, they would be recorded and reported at the end of the trial. Vital signs and allergic reaction signs were evaluated every 12 hours for all subjects.

### Ethical considerations

This study was approved by the Ethical Committee of Dr. Soetomo General Hospital (No. 1922/KEPK/11/2020, March 27 ^th^ 2020). Written informed consent obtained from the subjects’ parents or legal guardian. ****

### Participant allocation and blinding

**Figure 1.**
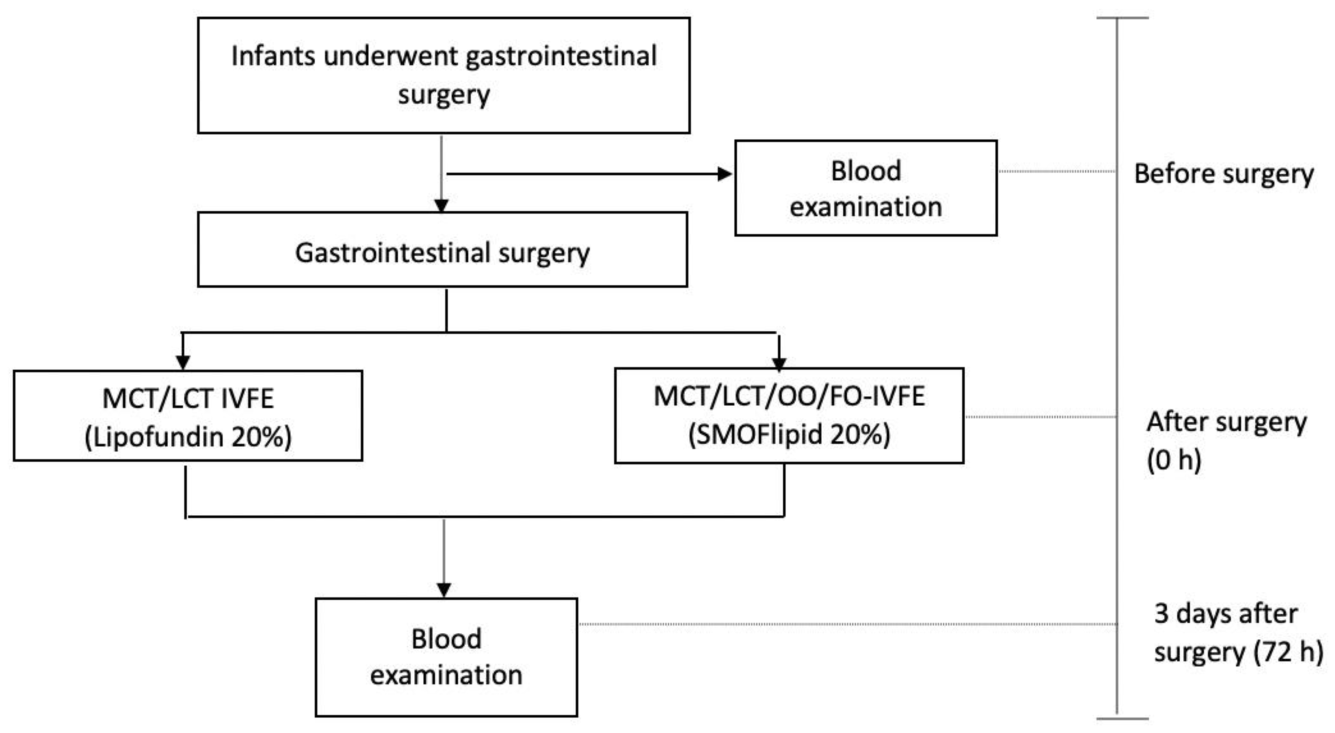
Study flowchart.

Subjects were randomly assigned to one of two IVLE groups following simple randomization procedures (computerized random numbers, https://www.random.org). Determination of whether a subject would get MCT/LCT standard IVLE or MCT/LCT/OO/FO was made by reference to a statistical series based on random sampling numbers drawn up by the primary investigator. Except the primary investigator and the pharmacist in charge, all subjects and staff were kept blind to IVLE assignment of the subjects. Twelve-folded numbered papers were placed into opaque sealed envelopes to be chosen by the subjects’ parents or legal guardian. Investigators and pharmacy staff opened the envelope and used the lipid emulsion assigned to that patient. The trial is registered at ClinicalTrials.gov, number NCT04511299, registered on August 13 ^th^ 2020. The protocol of this study can be seen at https://doi.org/10.17504/protocols.io.bknmkvc6. The flow of this research is shown on Figure 1.

### Interventions and measurement

The type of MCT/LCT IVLE used in this study was Lipofundin 20% which contained 50% coconut oil as the source of MCT and 50% soy oil as the source of LCT. Lipofundin 20% was given intravenously for three consecutive days after gastrointestinal surgery at 1–4 gram/kilogram/day dosing ^8^. The type of MCT/LCT/OO/FO IVLE used in this study was Smoflipid 20%, which contained 30% soy oil as the source of LCT, 30% coconut oil as the source of MCT, 25% olive oil, and 15% fish oil. SMOFlipid® was given for three consecutive days in 1–4 gram/kilogram/day dosing ^8^. The lipids used in this study were obtained from the manufacturers: Bbraun Indonesia (Lipofundin 20% I and Fresenius Kabi Indonesia (Smoflipid 20%). The comparison of the standard fat emulsion and ω-3-enriched fat emulsion is shown on Table 1. Both groups received isonitrogenous and isocaloric parenteral nutrition.

**Table 1.** Comparison of intravenous lipid emulsion content.

Oil source	Lipofundin 20% MCT/LCT	SMOF lipid 20%
Soybean oil, refined	10	6.0
Triglycerides, medium-chain	10	6.0
Olive oil, refined	0	5.0
Fish oil	0	3.0
Fat composition (g)		
Linoleic acid	2.7	2.9
Alpha-linolenic acid	0.4	0.3
Eicosapentaenoic acid	0	0.3
Docosahexaenoic acid	0	0.05
Oleic acid	1.1	2.8
Palmitic acid	0.7	0.9
Stearic acid	0.2	0.3
Arachidonic acid	0.02	0.05

Before surgery, blood samples of subjects were drawn (3–4 cm ^3^) in order to measure their IL-1β, IL-8 levels, fatty acid composition, also hemoglobin, leukocyte, CRP and albumin levels. After surgery, subjects were assigned to either MCT/LCT IVLE or MCT/LCT/OO/FO IVLE for three consecutive days. On 3 ^rd^ day (72 hours) after surgery, blood samples of subjects were drawn again in order to measure the same outcomes post-treatment. The blood samples were harvested after stopping infusion for 6 hours. Once the samples arrive to the laboratory, samples are allowed to clot for 30 minutes at room temperature before centrifugation for 15 minutes at 1000 x g. L-1β and IL-8 levels in the serum were examined using the Quantikine HS ELISA by R&D Systems (Catalog Number HSLB00D and HS800) at wavelengths of 540 nm and 650 nm, respectively. Fatty acid composition was analyzed using gas chromatography tandem mass spectrometry (GC-MS). This examination was measuring the levels of fatty acid in human serum quantitatively, including the arachidonic acid (AA)/EPA ratio, EPA, DHA, and AA. This method consists of two techniques, namely gas chromatography, which is a separation technique based on the degree of polarity and vapor point and mass spectrophotometry, which is a quadupole scanning spectrometer that can measure masses of 7-250 atomic mass units. Reagents used in this study were FAME Standard Mix (Supelco), GLC Nonadecanoic ISTD (Supelco), N-Hexane MS grade (Merck), Chloroform (Merck), Methanol Hyper Grade (Merck), Capillary Column and Helium Gas for GCMS. For this examination, once the samples arrive to the laboratory, samples are allowed to clot for 30 minutes at room temperature before centrifugation for 15 minutes at 1000 x g. After that, we made diluent by put 290 microliter of chloroform and 10 uL ISTD with concentration of 2.5 mg/ml to a serum cup. Add 100 microliter sample and 150 microliter methanol and let it homogenized with vortex for about 2 minutes. We added 300 microliter diluent sample and homogenized it for 2 minutes before centrifugation for 5 minutes at 2500 x g. After centrifugation, there were 3 layers. We took the bottom layer (chloroform containing analytes), dry the supernatant with nitrogen gradually, first 10 minutes at 5 Psi, and the next 30 minutes at 10 Psi. We redissolved it with 190 microliter n-Hexan and added 10 microliter of derivatization substances. After the serum cup were parafilmed and incubated for 2 hours, there were 2 layers. We took 100 microliter of top layer, added 400 microliter of n-Hexan to the GC vial amber, and injected it to the GCMS system. 

### Statistical analysis

Statistical analyses performed in this study were Mann-Whitney U-test, Fishers’ Exact test, independent sample t-test and chi-square test using SPSS for Mac 23.0. The analyses of the IL-1β, IL-8 levels, fatty acid composition, also hemoglobin, leukocyte, CRP and albumin levels were done by Mann-Whitney U-test or independent sample T-test as appropriate to test the significance between the two groups. A p-value less than or equal to 0.05 was considered statistically significant. The analysis of subjects’ characteristic were done by Mann-Whitney U-test, independent sample T-test, Fisher’s exact test or chi-square test as appropriate to test the significance between the two groups. All parameters examined were compared for differences before surgery, 3 days after surgery and differences within those 3 days (delta).

## Results

### Subject characteristics

**Figure 2.**
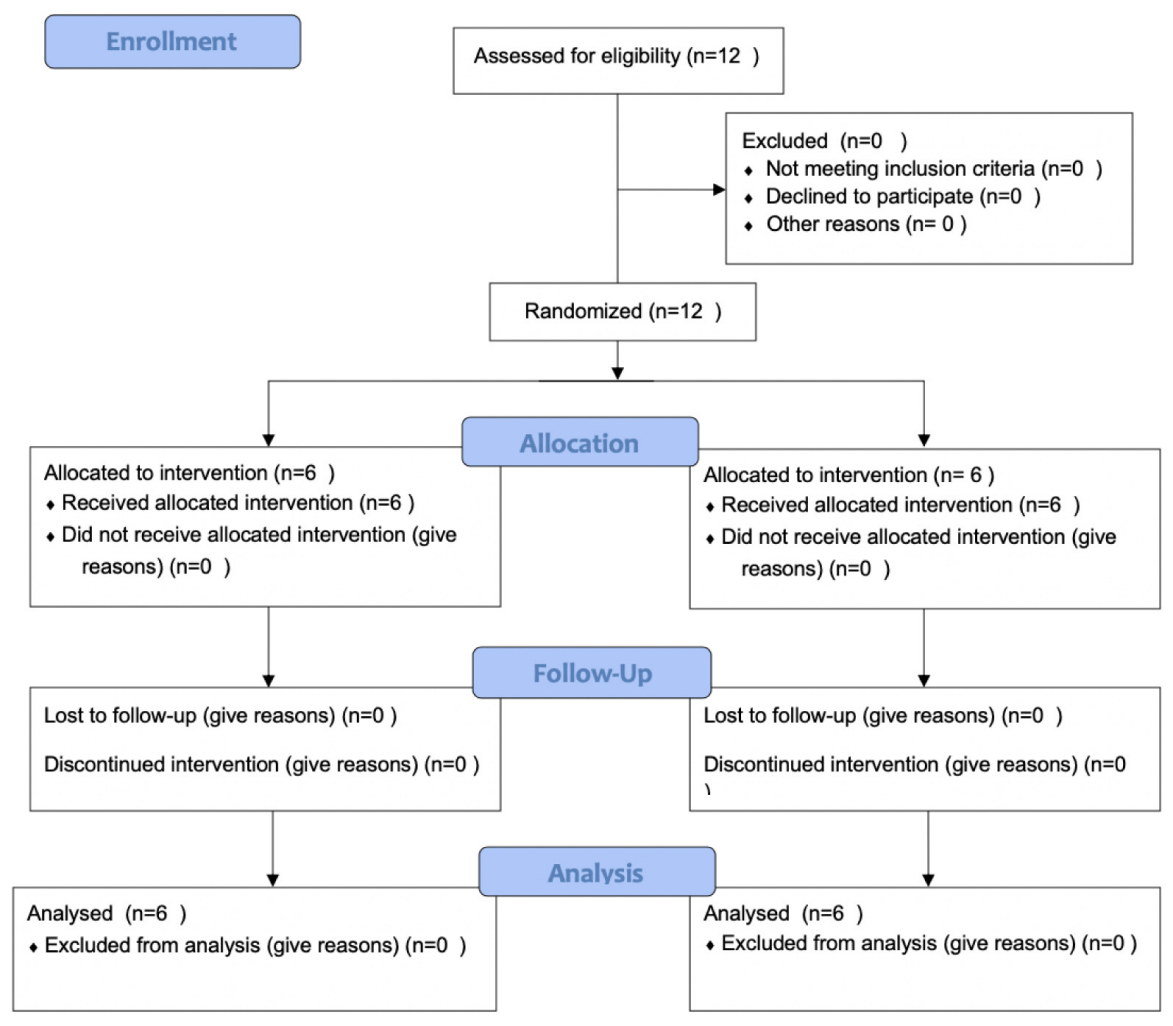
Study flow diagram.

This study enrolled 12 subjects at Soetomo General Hospital Surabaya who had undergone gastrointestinal surgery and met the inclusion and exclusion criteria. The recruitment flow of the subjects is shown on Figure 2. The subjects were classified into two groups: group 1 received intravenous MCT/LCT lipid emulsion, and group 2 received intravenous MCT/LCT/OO/FO lipid emulsion. Subject characteristics are shown in Table 2.

**Table 2.** Subject characteristics.

Characteristic	MCT/LCT	MCT/LCT/OO/FO	p-value
Age (days, median±IQR)	6.50±10.7	5.00±12.7	0.872 ^*^
Gender Male Female	3 (25%) 3 (25%)	5 (41.6%) 1 (8.4%)	0.545 ^**^
Birth weight (g, mean±SD)	2708.33±697.4	2681.67±497.2	0.941 ^***^
Diagnosis Anorectal malformation Pancreas annulare Oesophageal atresia Atresia duodenum	2 (16.6%) 1 (8.4%) 2 (16.6%) 1 (8.4%)	2 (16.6%) 3 (25%) 1 (8.4%) 0	0.506 ^****^

*Mann-Whitney test ** Fishers’ Exact test *** Independent Sample Test ****Chi-square test

No statistical difference was found in age, gender, birth weight, and diagnosis between both groups. De-identified subject characteristics, alongside all parameters measured in this study, are available as *Underlying data* ^19^.

### Primary outcomes

Mean IL-1β levels among subjects are depicted in Table 3; no difference was found in IL-1β levels between both groups before surgery (p = 0.873) and on day three after surgery (p = 0.873). We also did not find any difference in changes in IL-1β levels within 3 days (p = 0.906) in both groups.

**Table 3.** Cytokine levels in subjects.

Cytokine	MCT/LCT n=6			MCT/LCT/OO/FO n=6			P
	Before surgery	Day-3 post surgery	Changes within 3 days	Before surgery	Day-3 post surgery	Changes within 3 days	
L-1β (pg/ml, mean±SD)	3.65±5.3	3.84±4.6	0.19±5.6	1.93±1.6	2.42±1.5	0.49±1.9	0.873 ^*a^ 0.873 ^*b^ 0.906 ^**c^
IL-8 (pg/ml, mean±SD)	157.50±103.1	151.31±113.5	-6.20±171.5	130.01±109.4	144.46±110.5	14.4±166.5	0.688 ^*a^ 0.496 ^**b^ 0.837 ^**c^

*Independent sample t-test. **Mann-Whitney U-test. ^a^ Difference between groups before surgery. ^b^ Difference between groups 3 days post-surgery. ^c^ Difference between groups on changes within 3 days.

Furthermore, there was no significant difference in mean IL-8 levels between both groups before surgery (p = 0.688) and on day three after surgery (p = 0.494), and no difference in IL-8 levels changes within 3 days (p = 0.837) in both groups.

The analysis of fatty acid composition is shown on Table 4. No significant differences were observed in ω6/ω3 ratio, AA/EPA ratio, and EPA, DHA, and AA levels between both groups. Nevertheless, ω-6 fatty acids level was significantly lower in MCT/LCT/FO/OO IVLE group on third day after surgery group (p=0,048) compared to the standard IVLE.

**Table 4.** Serum fatty acid between standard and omega-3 enriched intravenous lipid emulsion.

Fatty acid (%total fatty acids, umol/L)	MCT/LCT N=6			ω-3 IVLE N=6			p-value
	Before surgery (Mean±SD)	Day-3 post surgery (Mean±SD)	Changes within 3 days (Mean±SD)	Before surgery (Mean±SD)	Day 3 post surgery (Mean±SD)	Changes within 3 days (Mean±SD)	
Ratio ω6/ω3	4.91±1.1	7.21±1.2	2.30±1.2	6.1±1.8	6.68±2.4	0.73±2.4	0.206 ^*^ 0.172 ^**^ 0.177 ^*^
AA/EPA	16.50±14.5	10.50±6.2	-6.00±15.1	83.83±100.3	15.67±16.9	-68.17±87.7	0.229 ^**^ 0.936 ^**^ 0.145 ^*^
ω 3	6.83±1.7	5.83±0.8	-1.00±1.4	5.51±1.7	5.83±1.5	0.33±1.0	0.214 ^*^ 0.739 ^**^ 0.092 ^*^
ALA	0.59±0.5	1.14±0.3	0.54±0.3	0.39±0.45	0.69±0.43	0.29±0.6	0.479 ^*^ 0.07 ^**^ 0.415 ^*^
EPA	0.99±0.6	3.42±1.4	-0.06±0.8	0.59±0.7	3.51±1.2	0.49±0.7	0.150 ^**^ 0.914 ^*^ 0.212 ^*^
DHA	5.35±1.6	3.86±0.6	-1.49±1.2	4.58±1.5	3.91±0.8	-0.67±1.3	0.412 ^*^ 0.907 ^*^ 0.270 ^*^
LA	20.29±10.3	32.77±2.8	12.47±7.9	19.45±8.8	26.65±6.2	7.20±10.6	0.882 ^*^ 0.053 ^*^ 0.352 ^*^
GLA	0.14±0.1	0.31±0.1	1.64±0.3	0.18±0.1	0.21±0.2	1.18±0.6	0.202 ^*^ 0.255 ^*^ 0.144 ^*^
DGLA	1.62±0.9	1.64±0.3	-0.23±0.6	1.71±0.7	1.18±0.6	-0.09±1.2	0.857 ^*^ 0.132 ^*^ 0.144 ^*^
AA	9.56±2.8	7.55±1.7	-1.68±1.9	10.71±4.3	7.69±2.5	-3.01±5.3	0.596 ^*^ 0.907 ^*^ 0.583 ^*^
ω-6	33.14±6.4	42.61±3.2	8.48±6.3	32.08±5.6	36.04±6.4	3.96±7.4	0.765 ^*^ 0.048 ^*^ 0.282 ^*^
OA	18.33±3.2	15.15±1.5	-3.18±2.9	17.71±5.2	17.38±3.9	-0.34±5.1	0.810 ^*^ 0.242 ^*^ 0.259 ^*^
MA	0.67±0.16	0.62±0.1	-0.06±0.1	0.79±0.3	0.74±0.3	-0.06±0.4	0.432 ^*^ 0.450 ^*^ 0.979 ^*^
PA	28.67±5.1	25.02±1.4	-3.65±4.6	30.75±5.4	28.30±3.8	-2.45±6.1	0.511 ^*^ 0.075 ^*^ 0.708 ^*^
SA	8.05±1.1	8.42±1.0	0.37±0.9	8.61±2.2	138.14±316.9	0.01±3.5	0.586 ^*^ 0.631 ^**^ 0.809 ^*^
Saturated	37.42±5.4	34.07±1.0	-3.36±4.7	40.16±6.6	37.66±3.5	-2.50±7.6	0.453 ^*^ 0.053 ^*^ 0.819 ^*^
MUFA	22.46±3.9	17.67±1.6	-4.79±2.95	22.18±5.3	20.56±5.4	-1.62±6.3	0.919 ^*^ 0.255 ^*^ 0.378 ^**^
PUFA	40.11±7.4	48.23±2.3	8.12±5.98	37.65±7.1	41.76±6.9	4.11±7.9	0.571 ^*^ 0.071 ^*^ 0.345 ^*^
Total	6509.50±1361.6	6309.34±1383.07	-200.17±1987.9	5175.34±3613.2	6414.17±1838.2	1238.84±3925.2	0.417 ^*^ 0.913 ^*^ 0.448 ^*^

*Independent Sample Test **Mann-Whitney Test AA: arachidonic acid, EPA: eicosapentaenoic acid, ALA: alpha-linolenic acid, DHA: docosapentaenoic Acid, LA: linoleic acid, GLA: gamma-linolenic acid, DGLA: dihomo-gamma-linolenic-acid, OA: oleic acid, MA: myristic acid, PA: palmitic acid, SA: stearic acid, MUFA: monounsaturated fatty acids, PUFA: polyunsaturated fatty acids.

### Secondary outcomes

The laboratory parameters are shown in Table 5. According to preoperative laboratory assessment, no statistical difference was found in hemoglobin, leukocyte, CRP and albumin levels between both groups. There was a statistically significant difference in leukocytes between both groups 3 days after surgery (p=0.025). CRP level was significantly lower in MCT/LCT/OO/FO group 3 days after surgery (p=0.01) and in changes within 3 days (p=0.016) compared to the standard MCT/LCT IVLE.

**Table 5.** Laboratory parameters in subjects.

Variable	MCT/LCT n=6			MCT/LCT/OO/FO n=6			P-value
	Before surgery	Day 3 Post surgery	Changes within 3 days	Before surgery	Day 3 Post surgery	Changes within 3 days	
Hemoglobin (g/dL)	15.65±2.2	12.78±1.8	-2.90±2.3	15.31±2.8	13.02±3.2	-2.30±1.6	0.826 ^*^ 0.879 ^*b^ 0.612 ^*c^
Leukocytes (/mm3)	17523.33±8362.7	13521.67±5121.5	-4001.67±8487.1	12788.67±5144.5	7653.33±1937.1	-511.34±6006.6	0.263 ^*a^ **0.025** ^*b^ 0.799 ^*c^
CRP (mg/dL)	0.28±0.3	4.23±7.1	3.95±7.2	2.61±2.5	0.38±0.4	-2.23±2.65	0.097 ^**a^ 0.010 ^**b^ **0.016** ^**c^
Albumin (g/dL)	3.20±0.4	3.12±0.5	-0.08±0.7	3.18±0.3	3.30±0.4	0.12±0.2	0,629 ^**a^ 0.493 ^**b^ 0.495 ^**c^

*Independent Sample Test **Mann-Whitney Test ^a^Differences between the MCT/LCT group and MCT/LCT/OO/FO before surgery ^b^ Differences between the MCT/LCT group and MCT/LCT/OO/FO 3 days post surgery ^c^ Differences between the MCT/LCT group and MCT/LCT/OO/FO in changes within 3 days

### Adverse effects

 There were no adverse effects reported from all subjects in the study.

## Discussion

A decrease in mean leukocyte levels in the ω-3-enriched IVLE group was observed on third day after surgery. This result is in accordance with some previous studies. Wei *et al*. observed significant declines in leukocyte and CRP levels on patients who received ω-3-enriched IVLE for 6 days after undergoing gastric tumor resection ^20^. Wang *et al*. also observed a significant decline in mean CRP level in subjects who received ω-3-enriched IVLE for 5 days after undergoing surgical interventions for acute pancreatitis ^21^. A systematic review stated that fish oil-enriched IVLE is associated with a reduction in CRP level in patients with malignancy after undergoing gastrointestinal surgery ^22^.

 The content of EPA and DHA in ω-3 lipids may inhibit inflammatory pathways in several ways, such as inhibiting chemotaxis of leukocytes, expression of adhesion molecules, and adhesive endothelial-leukocyte interaction ^16^.

 CRP is an acute-phase protein synthesized by IL-6 induction from hepatocytes. The CRP level spikes in acute traumatic condition, e.g. after undergoing surgical intervention. CRP levels reflect rapid changes which occur in inflammatory conditions. A study showed that in the majority of patients, CRP levels rise for 3-12 hours after surgery, peaking at 24-72 hours, and return to baseline in 2 weeks after surgery ^22^.

This study did not find any significant difference in IL-1β dan IL-8 levels between the two groups. This result is in accordance with some previous studies, including Ma *et al*. who did not find any difference in mean preoperative and postoperative (day-6) IL-1β levels between subjects who received intravenous MCT/LCT lipid emulsion and subjects who received intravenous MCT/LCT/OO/FO lipid emulsion for five consecutive days. Nevertheless, several studies yielded contrasting results ^23^.

A study by Wei *et al*. on 48 subjects who had undergone gastrointestinal tumor resection also found a significant reduction in mean IL-1β level on group receiving intravenous LCT/FO lipid emulsion compared to group receiving intravenous LCT lipid emulsion ^20^. In addition, Han *et al*., on 38 postoperative subjects in surgical intensive care unit, found a significant reduction in mean IL-1 and IL-8 levels on subjects who received MCT/LCT/FO lipid emulsion for 7 days, compared to subjects who received MCT/LCT lipid emulsion after undergoing surgical interventions ^24^.

A proposed explanation to why our result is inconsistent with most of the previous studies is that in our study, intravenous lipid emulsion was only given for 3 days, while in other studies, which yielded significant reduction in mean proinflammatory cytokine levels, intravenous lipid emulsion was given for at least 6 days. In this study, levels of IL-1β and IL-8 were examined preoperatively and 72 hours after surgery. According to Lin and Lowry, systemic inflammation, which occurs after surgery, may trigger proinflammatory and anti-inflammatory cytokines. Among all types of cytokines, TNF-α is the earliest to emerge, followed by IL-6 as the cytokine with the highest level amongst all. TNF-α and IL-6 levels peak in 1-2 hours after surgery ^25^. Our previous study showed a significant difference in mean IL-6 levels between subjects receiving MCT/LCT and subjects receiving MCT/LCT/OO/FO ^17^. Lin and Lowry stated that the half-life of IL-1β in systemic circulation is less than 10 minutes, making it more difficult to detect during stressful periods than TNF-α. Proinflammatory cytokine mediators, such as IL-8, are released as part of the inflammatory cascade initiated by IL-1 ^25^.

This study also observed that ω-6 fatty acids levels in the ω-3-enriched IVLE group was lower than the standard MCT/LCT IVLE group, on third day after surgery. This result is in accordance with several previous studies. Skouroliakou *et al*. found a significantly lower mean ω-6 fatty acids level in plasma fatty acid in preterm neonates who received ω-3-enriched IVLE for 15 and 30 days compared to soybean oil on the third day after abdominal surgery ^26^. Grim *et al*. showed a significant decline of ω-6 fatty acids levels in plasma phospholipids in 33 adult patients after major abdominal surgery who received ω-3-enriched fat emulsion for 6 days ^27^. The composition of fatty acids in cell membrane phospholipids has a significant role on cellular responses and cell function. Membrane order and lipid raft assembly are affected by the fatty acid makeup of membrane phospholipids. The fatty acid composition of the second messengers that are obtained from membrane phospholipid influences their biological activity and potency. Fatty acids that released from membrane phospholipids upon cellular activation are forming some lipid mediators ^28^. Nevertheless, our results on profiling of fatty acid in serum, such as ω6/ω3 ratio, EPA level, and DHA level, contradict previous studies which found a significant decline in ω-3-enriched fat emulsion group ^27, 29– 31^. This discrepancy might be due to dissimilarity of subjects’ characteristics, and the duration of parenteral nutrition administration. In those studies, the standard IVLE used were 100% LCT/soybean oil-based lipid emulsion, not MCT/LCT IVLE like in our study.

To our knowledge, this is the first study in Indonesia to compare the effect of MCT/LCT/OO/FO IVLE with MCT/LCT IVLE on proinflammatory IL-1β and IL-8 levels and fatty acid composition in infants who had underwent gastrointestinal surgery. Further studies are needed to determine the difference in pathogenesis between adults and infants after undergoing gastrointestinal surgery, which may be associated with the difference in effects of MCT/LCT/OO/FO IVLE on their profiling of fatty acid compositions.

There were no adverse event or serious adverse event reported in this study. Limitation of this study include that it is a single-centre study with a small sample size. Our study did not have a long period of follow-up and is not a double-blind study.

## Conclusion

In infants who underwent gastrointestinal surgery, MCT/LCT/OO/FO IVLE can significantly lower leukocyte, CRP and ω-6 fatty acids levels, and is comparable with standard IVLE on IL-1β & IL-8 levels.

## Data availability

### Underlying data

Figshare: Data Set Comparison of Two Lipid Emulsions on Interleukin-1β, Interleukin-8 and Fatty Acid Composition in Infants Post Gastrointestinal Surgery: A Randomized Trial. https://doi.org/10.6084/m9.figshare.12906320.v2 ^19^.

This project contains the underlying data for the study in SAV and CSV formats.

### Reporting guidelines

Figshare: CONSORT checklist for ‘Comparison of two lipid emulsions on interleukin-1β, interleukin-8 and fatty acid composition in infants post gastrointestinal surgery: a randomized trial’ https://doi.org/10.6084/m9.figshare.12906320.v4 ^32^.

Data are available under the terms of the Creative Commons Attribution 4.0 International license (CC-BY 4.0).
