# Supplementary material for: Comparison of two lipid emulsions on interleukin-1β, interleukin-8 and fatty acid composition in infants post gastrointestinal surgery: a randomized trial
Source: F1000Res. 2020 Dec 21;9:1168. Originally published 2020 Sep 24. [Version 3] doi: 10.12688/f1000research.26269.3 (PMC7707114; doi:10.12688/f1000research.26269.3)
Supplement: Peer review history

# Peer review: Reviewer response for version 3

**DOI:** 10.5256/f1000research.31417.r76333

**Published:** 2021-01-05

**Authors:** Philip C. Calder

**Affiliations:** 1 School of Human Development and Health, Faculty of Medicine, University of Southampton, Southampton, UK

This is a further revision of this article. The revisions improve the manuscript.

Is the work clearly and accurately presented and does it cite the current literature?

Yes

If applicable, is the statistical analysis and its interpretation appropriate?

Partly

Are all the source data underlying the results available to ensure full reproducibility?

Yes

Is the study design appropriate and is the work technically sound?

Yes

Are the conclusions drawn adequately supported by the results?

Yes

Are sufficient details of methods and analysis provided to allow replication by others?

No

Reviewer Expertise:

NA

I confirm that I have read this submission and believe that I have an appropriate level of expertise to confirm that it is of an acceptable scientific standard.

# Peer review: Reviewer response for version 3

**DOI:** 10.5256/f1000research.31417.r76335

**Published:** 2020-12-22

**Authors:** I Gusti Lanang Sidiartha

**Affiliations:** 1 Child Health Department, Faculty of Medicine, Sanglah General Hospital, Udayana University, Denpasar, Indonesia

I have read this revised version and I have no further comments to make.

 Thank you.

Is the work clearly and accurately presented and does it cite the current literature?

Yes

If applicable, is the statistical analysis and its interpretation appropriate?

Yes

Are all the source data underlying the results available to ensure full reproducibility?

Yes

Is the study design appropriate and is the work technically sound?

Yes

Are the conclusions drawn adequately supported by the results?

Yes

Are sufficient details of methods and analysis provided to allow replication by others?

Yes

Reviewer Expertise:

Pediatric Nutrition and Metabolic Disease

I confirm that I have read this submission and believe that I have an appropriate level of expertise to confirm that it is of an acceptable scientific standard.

# Peer review: Reviewer response for version 3

**DOI:** 10.5256/f1000research.31417.r76334

**Published:** 2020-12-22

**Authors:** Peter Stehle

**Affiliations:** 1 Department of Nutrition and Food Sciences, University of Bonn, Bonn, Germany

No further comments.

Is the work clearly and accurately presented and does it cite the current literature?

Yes

If applicable, is the statistical analysis and its interpretation appropriate?

Yes

Are all the source data underlying the results available to ensure full reproducibility?

Yes

Is the study design appropriate and is the work technically sound?

Yes

Are the conclusions drawn adequately supported by the results?

Yes

Are sufficient details of methods and analysis provided to allow replication by others?

Partly

Reviewer Expertise:

Clinical nutrition; nutritional physiology

I confirm that I have read this submission and believe that I have an appropriate level of expertise to confirm that it is of an acceptable scientific standard.

# Peer review: Reviewer response for version 2

**DOI:** 10.5256/f1000research.30813.r75205

**Published:** 2020-11-30

**Authors:** I Gusti Lanang Sidiartha

**Affiliations:** 1 Child Health Department, Faculty of Medicine, Sanglah General Hospital, Udayana University, Denpasar, Indonesia

PICO of this manuscript:

- P: infants who underwent gastrointestinal surgery.
- I: mix intravenous lipid emulsion, MCT/LCT/OO/FO.
- C: intravenous lipid emulsion, standard.
- O: IL-1b, IL-8, fatty acids.

Comments:

1. The assignment of patients to treatments was randomized.
2. The groups were similar at the start of the trial according to table 2.
3. Aside from the allocated treatment, the groups were treated equally.
4. All patients who entered the trial accounted for and randomized were analyzed at the end of the trial.
5. This trial used a single-blind where the participants did not know which treatment they received.
6. The treatment effect on IL-1b and IL-8 were changed by 0.19 and 6.20, respectively, while in the control group changes by 0.49 and 14.4, respectively. The results were not statistically significant with a p-value > 0.05.
7. The results will not help our patients.

So, this trial is valid but not important and applicable. It is needed to further study.

 My comment on this study is still that it is approved for indexing.

Is the work clearly and accurately presented and does it cite the current literature?

Yes

If applicable, is the statistical analysis and its interpretation appropriate?

Yes

Are all the source data underlying the results available to ensure full reproducibility?

Yes

Is the study design appropriate and is the work technically sound?

Yes

Are the conclusions drawn adequately supported by the results?

Yes

Are sufficient details of methods and analysis provided to allow replication by others?

Yes

Reviewer Expertise:

Pediatric Nutrition and Metabolic Disease

I confirm that I have read this submission and believe that I have an appropriate level of expertise to confirm that it is of an acceptable scientific standard.

# Peer review: Reviewer response for version 2

**DOI:** 10.5256/f1000research.30813.r75125

**Published:** 2020-11-23

**Authors:** Peter Stehle

**Affiliations:** 1 Department of Nutrition and Food Sciences, University of Bonn, Bonn, Germany

In general, the authors adequately answered my questions and modified the manuscript accordingly. Still not optimal is the definition of primary outcomes: following the strategy in power calculation, only interleukin 1ß can be "primary", all other parameters "secondary".

Is the work clearly and accurately presented and does it cite the current literature?

Yes

If applicable, is the statistical analysis and its interpretation appropriate?

Yes

Are all the source data underlying the results available to ensure full reproducibility?

Yes

Is the study design appropriate and is the work technically sound?

Yes

Are the conclusions drawn adequately supported by the results?

Yes

Are sufficient details of methods and analysis provided to allow replication by others?

Partly

Reviewer Expertise:

Clinical nutrition; nutritional physiology

I confirm that I have read this submission and believe that I have an appropriate level of expertise to confirm that it is of an acceptable scientific standard.

# Peer review: Reviewer response for version 2

**DOI:** 10.5256/f1000research.30813.r75124

**Published:** 2020-11-23

**Authors:** Philip C. Calder

**Affiliations:** 1 School of Human Development and Health, Faculty of Medicine, University of Southampton, Southampton, UK

This is the revised version of this manuscript. The authors have taken the previous comments seriously and have made significant revisions. These certainly improve the manuscript. However, some minor points for correction remain (see below). In addition there are serious issues with a) the power calculation and b) the fatty acid data.

1. Throughout the manuscript w-6 and w-3 should be followed by "fatty acids".
2. Abstract. Background refers to "plasma fatty acids" but these were determined in serum.
3. Abstract conclusion. "infants underwent" should read "infants who had undergone".
4. The power calculation is seriously flawed. We are told that an effect on IL-1b was used to power the study. An effect size of 20 pg/ml was used; there is no indication of what that effect size or the SD is based on. Effect size is the estimated size of the effect that will occur in the treatment group compared to the control (in other words a decrease of 20 pg/ml). However IL-1b at study entry was 3.65 pg/ml. Thus it is impossible to have an effect size of 20 pg/ml in this cohort.  
5. Patient allocation. We are told that 8 numbered papers were put in envelopes and removed at random. However there are 12 participants. It is not clear how 8 numbers can allocate 12 participants.
6. The authors provide more detail on the fatty acid analysis which is welcome (but see later points). They use the word "deconstructed" to describe one of their steps. This word is absolutely important in order to understand what they have done. Do they actually mean the lipid has been "deconstructed" i.e. somehow broken up? Or do they simply mean it was "redissolved" in solvent.
7. The fatty acid standard is added in concentration units of mg/ml. However the fatty acid data are given in units of umol/l. How was this transformation done?
8. CRP is 10 x higher in one group than the other at study entry. Why?
9. Fatty acid data. ALA increased from 0.39 to 18.35 but the change between these values is 0.29. How can that be?
10. "Free" fatty acid data. I still have an issue understanding these data. Total "Free" fatty acids is shown at about 6500 umol/l. This is ten times higher than values quoted for "non-esterified" (i.e. "free") fatty acids elsewhere and does not seem credible. I wonder whether the authors are actually reporting "total" fatty acids (i.e. the sum of fatty acids in triglycerides, cholesteryl esters, phospholipids and free). This is a vital point that needs clarifying as without being clear about this it is impossible to understand these data. This also relates back to my question about the word "deconstructed".

Is the work clearly and accurately presented and does it cite the current literature?

Yes

If applicable, is the statistical analysis and its interpretation appropriate?

Partly

Are all the source data underlying the results available to ensure full reproducibility?

Yes

Is the study design appropriate and is the work technically sound?

Yes

Are the conclusions drawn adequately supported by the results?

Yes

Are sufficient details of methods and analysis provided to allow replication by others?

No

Reviewer Expertise:

Fatty acids; Human nutrition; Inflammation and immunity; Artificial nutrition support; Clinical trials

I confirm that I have read this submission and believe that I have an appropriate level of expertise to state that I do not consider it to be of an acceptable scientific standard, for reasons outlined above.

# Response

**Published:** 2020-12-11

**Authors:** Meta Herdiana Hanindita

**Affiliations:** Universitas Airlangga, Indonesia

Thank you again for your willingness to re-review this article. There are some changes made based on your suggestions. These are the responses to your previous comments.

1. The words “fatty acids” were already added throughout the manuscript.
2. It’s already changed to “serum fatty acids”.
3. It’s already revised to “infants who had undergone”.
4. We decided to use the effect size of 20 pg/ml based on this research: https://doi.org/10.1016/j.clnu.2018.06.929. There is no research on IL-1b level in infant underwent gastrointestinal surgery before. The closest sample to our research is this one. I hope that explains why we decided to use 20 pg/ml as our effect size. 
5. It’s already revised to twelve-numbered papers.
6. We simply mean it was re-dissolved (already changed).
7. The conversion from mg/ml to umol/l was done with molar mass/mass (molecular weight).
8. We believed that it could be caused by huge variation due to our small sample size. (It's our study's limitation).
9. Thank you for pointing this out. We rechecked our dataset and found an honest mistake. One data in ALA post-surgery was written as 107.0 which should be written as 1.07. We made a revised dataset and recounted the statistic analysis.
10. What is measured is the sum of fatty acids in triglycerides, cholesteryl esters, phospholipids, and free.

# Peer review: Reviewer response for version 1

**DOI:** 10.5256/f1000research.28994.r71909

**Published:** 2020-10-06

**Authors:** Peter Stehle

**Affiliations:** 1 Department of Nutrition and Food Sciences, University of Bonn, Bonn, Germany

This manuscript summarizes results gained within a clinical nutrition trial in infants after gastrointestinal surgery. Infants parenterally received either a "traditional", omega 6-rich lipid emulsion composed of soybean oil and MCT, in the "verum" group a newly composed lipid emulsion produced with a blend of MCT, olive oil, fish oil and some soybean oil was administered. The study was generally well designed and performed; data presentation and interpretation is adequate. However, some information should be added and/or sharpened.

1. Power calculation: Which parameter was used to make calculations? One of the primary outcomes? And which (already published) data were considered (sensitivity of the method, expected changes in concentrations)? Very crucial: Is that power calculation also valid for fatty acid analyses?
2. General nutritional concept: Which other components (glucose, amino acids...) were administered? And in which amounts? Was the concept isonitrogenous and isoenergetic?
3. What was the reason to give lipids for (only) 3 consecutive days?
4. Analysis of fatty acids in blood samples: It is unclear what has been measured: only "free" fatty acids? Fatty acid composition of (total) lipids? When free fatty acids have been measured: how long after stopping infusion the blood sample was harvested? Please, comment and complete the text.
5. Table 4: The capture is misleading. Please, reword and include that these data are "blood" analyses (see 4.).
6. Discussion: Any comparison with previous analytical data with respect to fatty acid profiles should mention what was measured (free fatty acids, lipid composition etc.) and under which clinical conditions.
7. Discussion: As mentioned by the authors themselves, metabolites, e.g. cytokines, are endogenously synthesized from fatty acid precursors released from membranes. With this background: how should a 3 day-infusion influence these metabolite concentrations? 

     

Is the work clearly and accurately presented and does it cite the current literature?

Yes

If applicable, is the statistical analysis and its interpretation appropriate?

Yes

Are all the source data underlying the results available to ensure full reproducibility?

Yes

Is the study design appropriate and is the work technically sound?

Yes

Are the conclusions drawn adequately supported by the results?

Yes

Are sufficient details of methods and analysis provided to allow replication by others?

Partly

Reviewer Expertise:

Clinical nutrition; nutritional physiology

I confirm that I have read this submission and believe that I have an appropriate level of expertise to confirm that it is of an acceptable scientific standard, however I have significant reservations, as outlined above.

# Response

**Published:** 2020-11-14

**Authors:** Meta Herdiana Hanindita

**Affiliations:** Universitas Airlangga, Indonesia

Thank you for your willingness to review this paper. 

 1. Details on the power calculation are already added in the paper. The parameter that  was used for sample size calculation was Interleukin-1b, with effect size of 20 pg/ml, and SD of 10.6 pg/ml. The formula used datas from our previous published research (reference no.17). No, the power calculation is only valid for interleukin-1b. 

 2. Yes, the concept was isonitrogenous and isocaloric. This statement is already added to the paper. 

 3. Our consideration for giving lipids for 3 consecutive days was because we wanted to know the inflammatory response in the initial/early inflammatory phase in the postoperative wound healing period. The initial effect of inflammation in the postoperative wound healing period occurs within 0-3 days.

 4. It should be written as serum free fatty acid (revised already). Details on this were already included in the paper. The blood samples were harvested after stopping infusion for 3 hours.

 5. It is already changed. 

 6. It is already added. 

 7. This study is the first study in Indonesia to examine the effects of intravenous fat emulsions on metabolites (such as cytokines) in postoperative infants. To be honest, we are making this study the baseline for our future studies. 3 days was chosen because this is the initial inflammatory period in the wound healing period. Looking at the results of this study, we probably will make further studies using lipid emulsion in infants after surgery over a longer time such as 7 days, 14 days and more than 14 days.

# Peer review: Reviewer response for version 1

**DOI:** 10.5256/f1000research.28994.r71910

**Published:** 2020-09-28

**Authors:** Philip C. Calder

**Affiliations:** 1 School of Human Development and Health, Faculty of Medicine, University of Southampton, Southampton, UK

This manuscript describes findings from a clinical trial in neonates who underwent surgery and then were randomised to receive one of two lipid emulsions intravenously over the course of 3 days. One lipid emulsion was a mix of coconut and soybean oils (control) and the other was a mix of coconut, soybean, olive and fish oils (treatment). The main outcomes were two cytokines (IL-1b and IL-8) and serum fatty acids. In addition a number of secondary outcomes are reported including white cell count and CRP. This study is of interest. A major limitation is sample size: there were 6 infants per group. The cytokines were not different between groups, but white cells and CRP were lower and omega-6 fatty acids higher at 3 days in the treatment group. The article is well written, data are clearly presented and the discussion refers to relevant existing literature.

 Comments:

1. I believe the abstract should report findings for serum omega-3 fatty acids.
2. For the sample size calculation what outcome(s) was used? This is not stated. Usually an effect size in a specific outcome is needed to calculate sample size.
3. Table 1. Lists docosapentaenoic acid but this should read eicosapentaenoic acid.
4. More detail is required about the fatty acid analysis. In the methods section you refer to “free fatty acids in serum” but in the Discussion you refer to “profiling phospholipid fatty acids”. It is necessary to a) clarify what you have actually measured and b) provide details of how the serum was processed prior to gas chromatography.
5. Stat analysis section is written in future tense (will be) but should be in the past tese (were/was).
6. Stat analysis. The key comparison is between groups either at day 3 controlling for baseline or the change to day 3. It is not clear whether this comparison has been done.
7. Data display. What are the errors shown: SD or SEM?
8. Data that does nth ae normal distribution should not be shown as mean and SD/SEM but as median and IQR.
9. Table 4. What are the units for total fatty acids?
10. Table 4. Huge variation is apparent here bringing the small sample size into focus. For example in the treatment group ALA changes from 0.39 pre to 18.35 post but this almost 50-fold increase is not significant.
11. Table 4. Why does EPA increase in the MCT/LCT group?

Is the work clearly and accurately presented and does it cite the current literature?

Yes

If applicable, is the statistical analysis and its interpretation appropriate?

Partly

Are all the source data underlying the results available to ensure full reproducibility?

Yes

Is the study design appropriate and is the work technically sound?

Yes

Are the conclusions drawn adequately supported by the results?

Yes

Are sufficient details of methods and analysis provided to allow replication by others?

No

Reviewer Expertise:

Fatty acids; Human nutrition; Inflammation and immunity; Artificial nutrition support; Clinical trials

I confirm that I have read this submission and believe that I have an appropriate level of expertise to state that I do not consider it to be of an acceptable scientific standard, for reasons outlined above.

# Response

**Published:** 2020-11-14

**Authors:** Meta Herdiana Hanindita

**Affiliations:** Universitas Airlangga, Indonesia

Thank you for your willingness to review this paper, it's a great honour for us. 

 1. Serum omega-3 fatty acid finding is already included in the abstract. 

 2. For the sample size calculation, the outcome used was Interleukin-1beta. Detail on this sample size calculation is already added in the paper. The effect size was 20 pg/ml, with SD 10.6 pg/ml. 

 3.Table 1 is already revised. 

 4. Detail on the fatty acid analysis is already added. It should be serum free fatty acid. 

 5. Stat analysis section is already revised.

 6. All parameters examined were compared for differences before surgery, 3 days after surgery (controlling to baseline) and differences within those 3 days (delta). This statement is already added to the paper. 

 7. The errors shown in SD. 

 8. We changed the data that does not have normal distribution, is already shown in median and IQR. 

 9. The unit is umol/L (added).

 10. Yes, unfortunately due to limitation of this study (small sample size). The incidence of these cases in infants in our hospital that needed parenteral nutrition was only 18-20 patients/year. 

 11. We really tried to find reasons for this finding but we could not find one. We need to have another research on this, to understand its mechanism better.
